# Chiral phosphoric acid-catalyzed asymmetric synthesis of helically chiral, planarly chiral and inherently chiral molecules

**DOI:** 10.3762/bjoc.21.145

**Published:** 2025-09-10

**Authors:** Wei Liu, Xiaoyu Yang

**Affiliations:** 1 Key Laboratory of Subcritical High-Efficiency Extraction, College of Chemistry and Environmental Engineering, Anyang Institute of Technology, Anyang, Henan 455000, China; 2 School of Physical Science and Technology, ShanghaiTech University, Shanghai 201210, China

**Keywords:** asymmetric catalysis, chiral phosphoric acid, helical chirality, inherent chirality, planar chirality

## Abstract

Chiral molecules, distinguished by nonsuperimposability with their mirror image, play crucial roles across diverse research fields. Molecular chirality is conventionally categorized into the following types: central chirality, axial chirality, planar chirality and helical chirality, along with the more recently introduced inherent chirality. As one of the most prominent chiral organocatalysts, chiral phosphoric acid (CPA) catalysis has proven highly effective in synthesizing centrally and axially chiral molecules. However, its potential in the asymmetric construction of other types of molecular chirality has been investigated comparatively less. This Review provides a comprehensive overview of the recent emerging advancements in asymmetric synthesis of planarly chiral, helically chiral and inherently chiral molecules using CPA catalysis, while offering insights into future developments within this domain.

## Introduction

**Figure 1:**
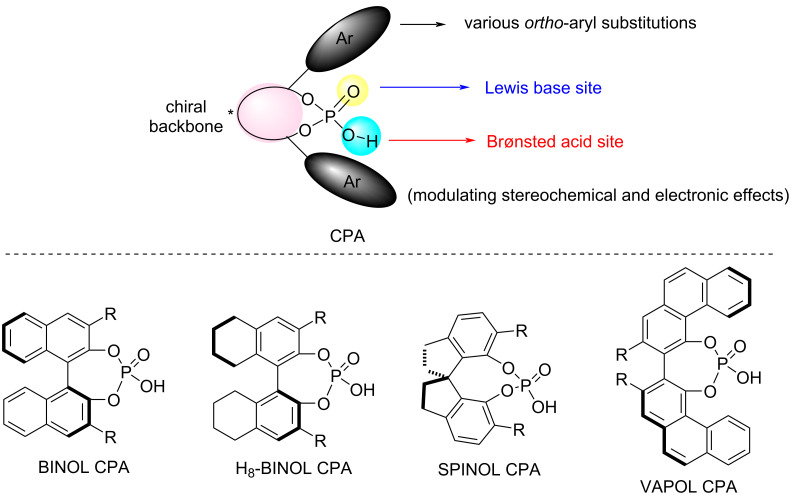
General structure of CPAs and selected CPAs with various chiral scaffolds.

Since the seminal works by Akiyama [1] and Terada [2] et al. in 2004 demonstrated the application of BINOL-derived chiral phosphoric acids (CPAs) in asymmetric Mannich reactions, the past two decades have witnessed the remarkable evolution of CPA catalysis into one of the most versatile platforms for achieving diverse enantioselective transformations [3–8]. CPA catalysts are generally recognized as bifunctional catalysts with two distinct catalytic sites. The OH group on the phosphorus atom functions as a Brønsted acid site, while P=O serves as a Lewis base site, which enables the simultaneous activation of both nucleophiles and electrophiles in one reaction (Figure 1). The chiral properties of the catalysts are derived from the chiral framework of the diol precursors, predominantly axially chiral structures such as BINOL, H_8_-BINOL, SPINOL and VAPOL scaffolds, which are widely used in the development of CPA catalysts. Furthermore, the *ortho*-aryl substitutions of the CPA catalyst can efficiently modulate the stereochemical and electronic effects of the CPAs, which establish a chiral microenvironment within the chiral scaffold that governs the stereoselectivity of asymmetric reactions.

**Figure 2:**
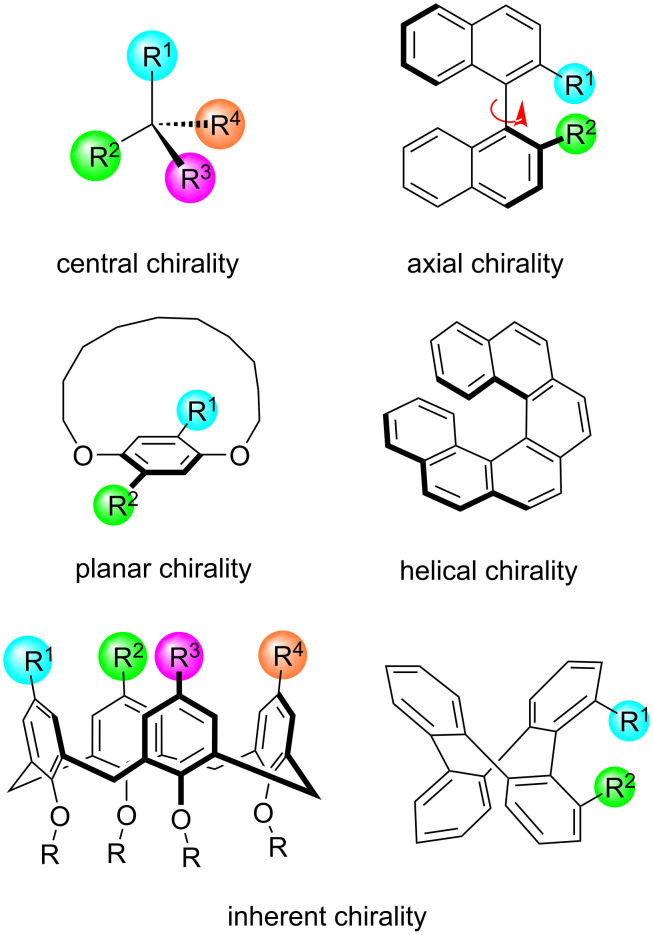
Representative elements of molecular chirality.

Chiral molecules, characterized as three-dimensional structures that are nonsuperimposable with their mirror image, have significant applications in pharmaceutical, agrochemical and asymmetric synthesis as well as materials science, to name a few examples. Molecular chirality is typically classified into four types of chiral elements: central (point) chirality, axial chirality, planar chirality and helical chirality (Figure 2). Moreover, unique forms of chirality originating from the rigid conformation of molecules lacking symmetry, which do not fit into the aforementioned four categories, are termed inherent chirality. Notable examples include inherently chiral calix[4]arenes and saddle-shaped medium-sized cyclic compounds. Catalytic asymmetric synthesis has been recognized as the most straightforward and efficient strategy for synthesizing chiral molecules, with early development primarily targeting compounds featuring stereogenic centers. In the past decade, significant progress has been made in the asymmetric synthesis of diverse axially chiral molecules [9]. However, the exploration of catalytic asymmetric synthesis toward other forms of chiral elements has been relatively limited, with only a few notable instances having emerged recently.

Similarly, since the initial introduction of CPA catalysts in asymmetric synthesis in 2004, a plethora of asymmetric catalytic reactions to synthesize chiral molecules with stereogenic centers has been developed. Moreover, the rapid advancement of axially chiral molecules in asymmetric synthesis has been made possible by employing CPA catalysis, notably pioneered by Akiyama [10], Tan [11] and others. However, the application of CPA catalysis in the asymmetric synthesis of other forms of molecular chirality has received less attention. While List and co-workers reported the first CPA-catalyzed asymmetric synthesis of helically chiral azahelicenes through the Fischer indole synthesis back in 2014 [12], the second CPA-catalyzed asymmetric synthesis of helicenes was not achieved until 2023 [13–14]. Similarly, the CPA-catalyzed asymmetric synthesis of planarly chiral [15] and inherently chiral [16] molecules was not disclosed until 2022. In this Review, we have comprehensively summarized the recent advancements in the CPA-catalyzed asymmetric synthesis of various distinct chiral elements, encompassing helically, planarly and inherently chiral molecules. The Review is structured based on the various types of chiral elements, presenting a representative substrate scope for each method, showcasing the reaction mechanisms and applications of the chiral products for selected examples.

## Review

### Helical chirality

Helicenes are a group of rigid polycyclic aromatic compounds composed of *ortho*-fused aromatic (hetero)cyclic rings, with their helically twisted conformation enforced by steric hindrance between terminal aromatic rings [17]. Despite lacking asymmetric stereogenic centers, this nonplanar scaffold exhibits intrinsic *P*/*M* chirality due to the helical arrangement of the π-extended skeleton. Renowned for their high thermal stability and structural rigidity, chiral helicenes have emerged as prominent molecular platforms in various applications, such as circularly polarized luminescence (CPL) materials, chiral liquid crystals and asymmetric catalysis. Currently, the asymmetric catalytic synthesis of helicenes predominantly revolves around transition metal-catalyzed asymmetric annulation reactions, including the asymmetric [2 + 2 + 2] cycloaddition of aryl-substituted polyynes and hydroarylation of alkynes [18–19]. In contrast, the application of asymmetric organocatalysis for enantioselective synthesis of chiral helicenes remains relatively underdeveloped compared to transition metal-catalyzed approaches [20].

**Scheme 1:**
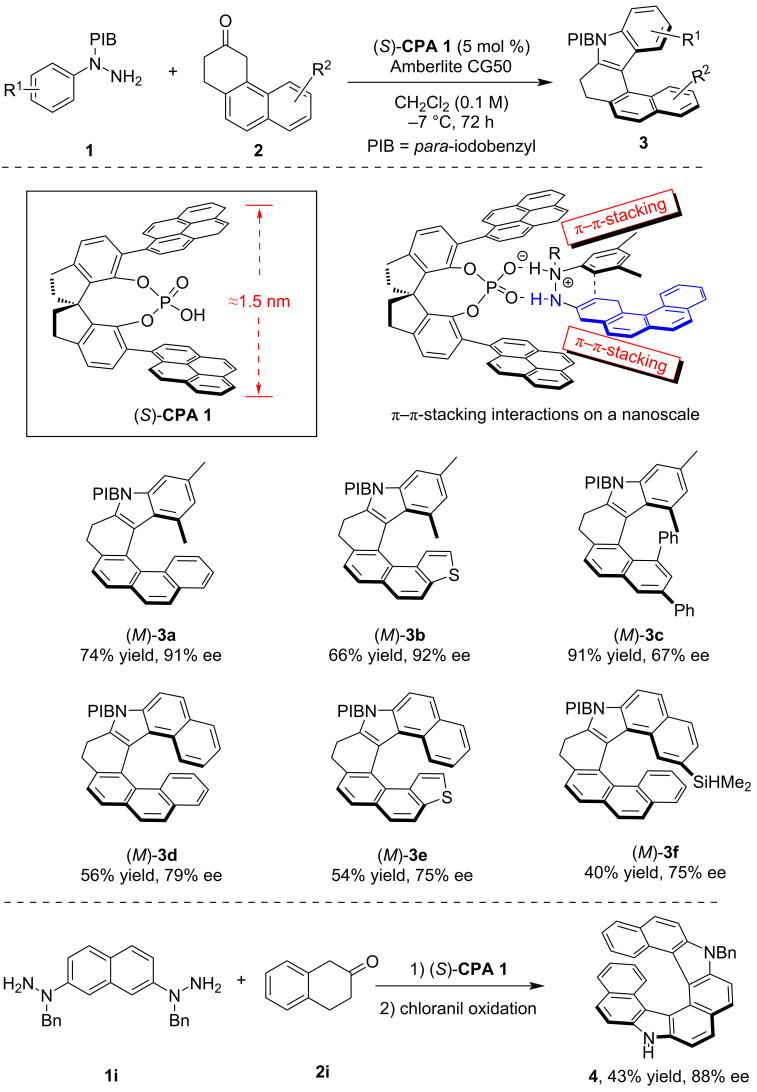
CPA-catalyzed asymmetric synthesis of azahelicenes via Fischer indole synthesis.

In 2014, List and co-workers reported the pioneering CPA-catalyzed asymmetric synthesis of helically chiral molecules, which also marked the first organocatalyzed asymmetric synthesis of such compounds [12]. By employing a CPA-catalyzed asymmetric Fischer indolization reaction of hydrazine **1** and polycyclic ketone **2**, they achieved the efficient asymmetric synthesis of various helically chiral azahelicenes **3** (Scheme 1). To address the inherent length-scale challenges of molecular helicene frameworks, the authors designed and synthesized novel CPAs bearing extended π-substituents at the *ortho*-positions. The dual hydrogen-bonding interactions were critical for this reaction, ensuring that the reaction proceeded within the chiral pocket of the CPA catalyst. Moreover, the authors proposed that the extended π-substituents at the *ortho*-positions of CPA could engage in π–π-stacking interactions with the enehydrazine intermediate, which is essential for achieving high levels of stereocontrol. Using the optimal catalyst **CPA 1**, a series of aza[6]helicenes **3a**,**b** was synthesized with excellent enantioselectivity and high yield. However, this method demonstrated notably reduced efficiency and stereoselectivity for the more sterically demanding aza[5]helicene **3c** and aza[7]helicenes **3d–f**. Furthermore, the authors expanded this methodology to a double Fischer indolization reaction between hydrazine **1i** and ketone **2i**, which yielded diaza[8]helicene **4** with moderate yield and high enantioselectivity after chloranil-mediated dehydrogenation.

**Scheme 2:**
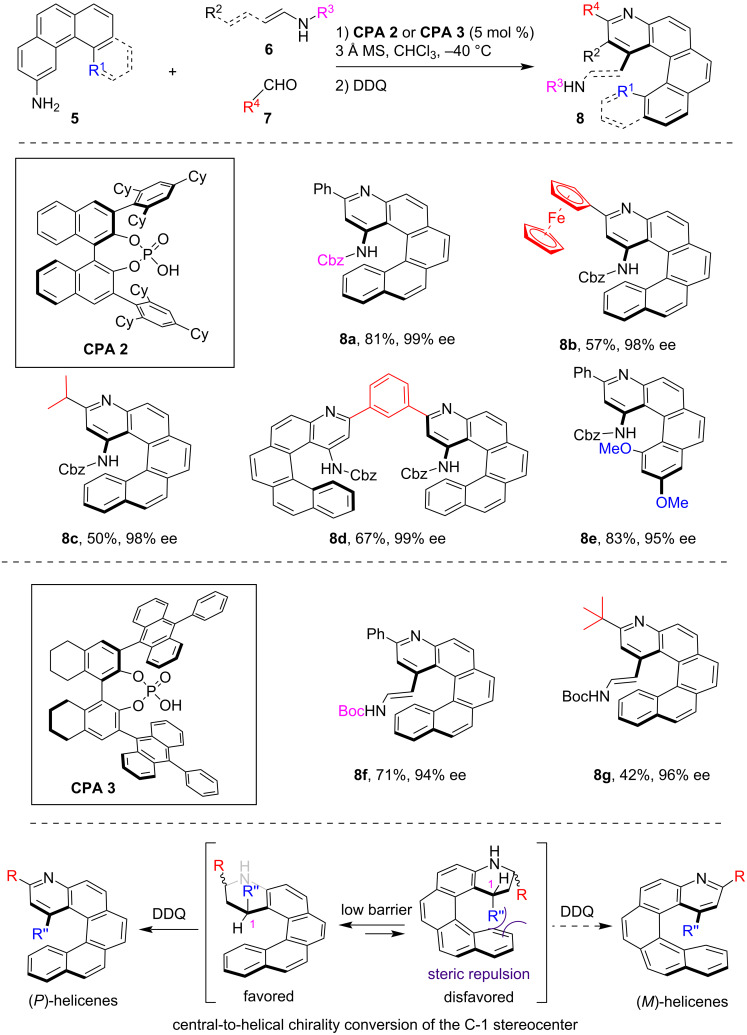
CPA-catalyzed asymmetric synthesis of azahelicenes via sequential Povarov reaction and oxidative aromatization.

Despite the early demonstration of CPA catalysis in synthesizing chiral helicenes, the next instance of CPA-catalyzed asymmetric synthesis of helicenes was not achieved until 2023. Employing a sequential CPA-catalyzed asymmetric Povarov reaction and oxidative aromatization process, in 2023 our group reported the asymmetric synthesis of various azahelicenes **8** from polycyclic arylamines **5**, dienamides **6** and aldehydes **7** (Scheme 2) [13]. This methodology demonstrates a broad substrate scope, enabling the efficient asymmetric synthesis of diverse aza[5]helicenes **8a–d** and aza[4]helicene **8e** from various aldehydes with high enantioselectivity. In addition, dienamides were found to be compatible with this method, albeit requiring a switch to **CPA 3** as the optimal catalyst, which generated the 1-enamide-substituted azahelicenes **8f**,**g**, with significant potential for diverse derivatizations. Based on experimental and computational studies, the origin of helical chirality in this method was elucidated. We proposed that the asymmetric Povarov reaction would generate a pair of diastereomeric tetrahydroquinoline derivatives displaying helical conformation, with a modest energetic barrier for interconversion. However, steric repulsion between the C-1 substitutions and the terminal arene moieties in the *M*-conformational diastereomer resulted in the *P*-conformational diastereomer being thermodynamically favored. This led to the formation of (*P*)-helicene products following DDQ-mediated dehydrogenation.

**Scheme 3:**
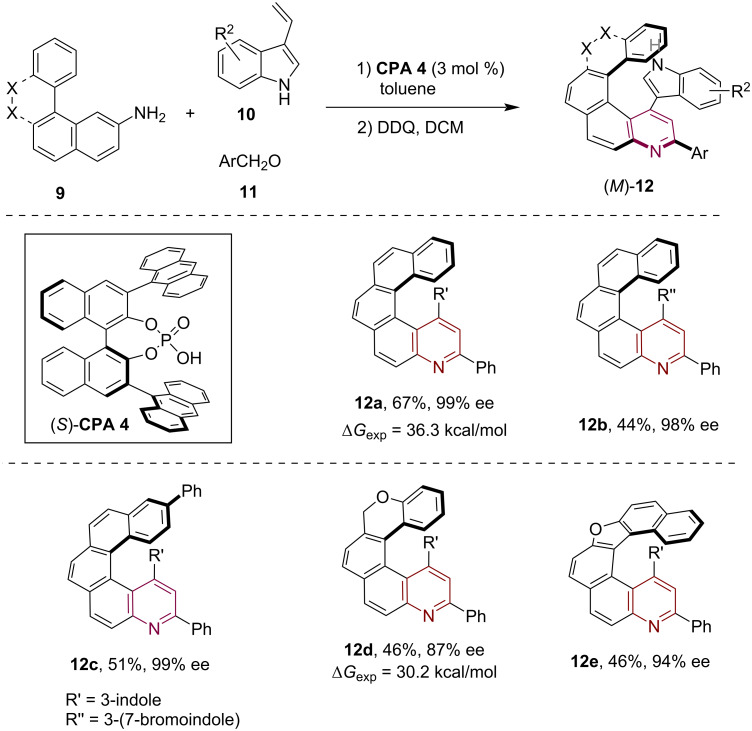
CPA-catalyzed asymmetric synthesis of azahelicenes via sequential Povarov reaction involving 3-vinylindoles and oxidative aromatization.

Almost simultaneously, the Li group independently reported the asymmetric synthesis of chiral quinohelicenes using a similar sequential asymmetric Povarov reaction and oxidative aromatization strategy [14]. In their study, they employed 3-vinylindoles **10** in the CPA-catalyzed asymmetric Povarov reaction with polycyclic arylamines **9** and various aromatic aldehydes **11**, resulting in a range of quinoline-containing azahelicenes **12** with moderate yield and excellent enantioselectivity after DDQ-mediated oxidative aromatization (Scheme 3). Notably, they not only expanded the substrate scope to encompass various aldehydes and 3-vinylindoles but also conducted extensive structural modifications on the polycyclic arylamine components, which enabled the asymmetric synthesis of azahelicenes with diverse frameworks, including the chromene- and furan-containing quinohelicenes **12d**,**e**, respectively. They also conducted a thorough evaluation of the stability of the helical chirality across the synthesized quinohelicenes, indicating a high racemization barrier.

**Scheme 4:**
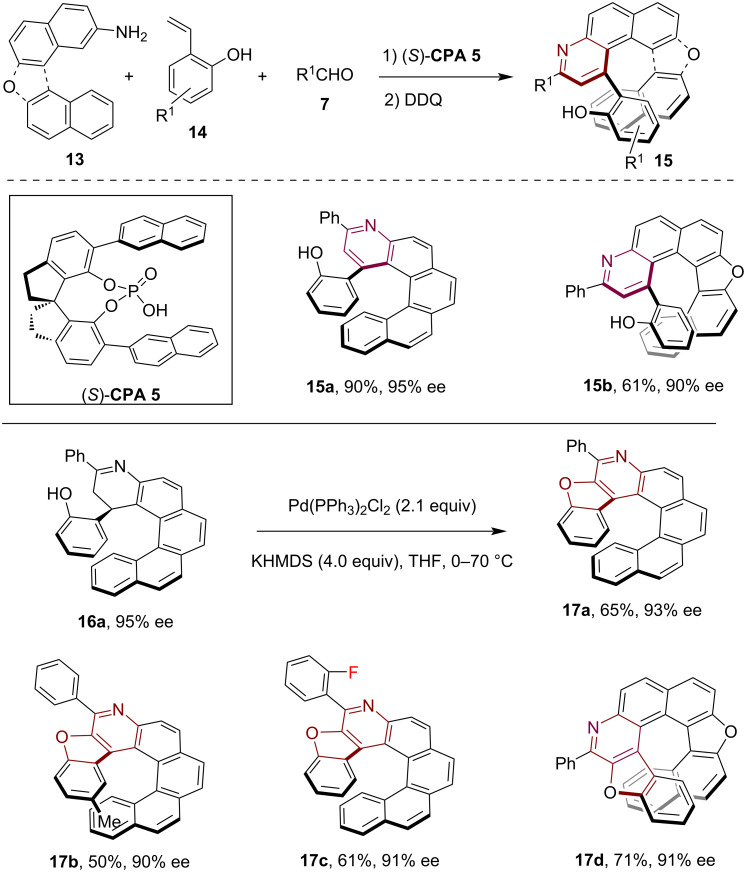
CPA-catalyzed asymmetric synthesis of heterohelicenes via sequential Povarov reaction involving 2-vinylphenols and aromatization.

In 2024, our group further extended the CPA-catalyzed sequential Povarov reaction and aromatization strategy by using 2-vinylphenols **14** as the olefin component, which facilitated the asymmetric synthesis of substituted [5]- and [6]pyridohelicenes **15** with *ortho*-phenolic substituents in position C1 with high enantioselectivity (Scheme 4) [21]. Notably, utilizing one equivalent of DDQ for semioxidation of the tetrahydroquinoline product of the Povarov reaction produced the imine **16a** which, upon treatment with Pd(PPh_3_)_2_Cl_2_ and KHMDS, led to furan ring formation and the generation of hetero[7]helicene **17a** while maintaining the stereochemical configuration. Through this methodology, a range of elongated [7]- and [8]heterohelicenes **17b**–**d** incorporating both furan and pyridine moieties were successfully synthesized with high enantioselectivity. These compounds would be challenging to access using alternative asymmetric methods.

**Scheme 5:**
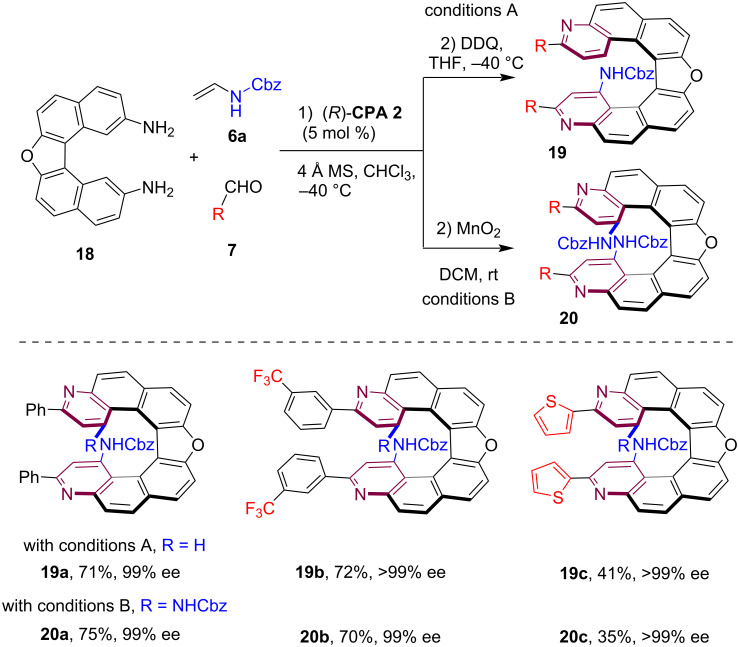
Diverse enantioselective synthesis of hetero[7]helicenes via a CPA-catalyzed double annulation strategy.

In 2025, our group disclosed a highly efficient catalytic enantioselective double annulation approach for the asymmetric synthesis of hetero[7]helicenes [22]. By employing a sequential CPA-catalyzed three-component double Povarov reaction involving a pentacyclic diamine substrate **18**, enamide **6a** and various aldehydes **7**, followed by oxidative aromatization, a range of bispyridine-containing hetero[7]helicenes was produced with good yield and excellent enantioselectivity (Scheme 5). Notably, two distinct oxidative aromatization methods have been developed to yield diverse heterohelicene products. For instance, using DDQ as an oxidant selectively delivered hetero[7]helicenes **19** with monoamido substitution at the *peri*-positions, while utilizing MnO_2_ as an oxidant selectively yielded heterohelicenes **20** with bisamido substitution at the *peri*-positions.

**Scheme 6:**
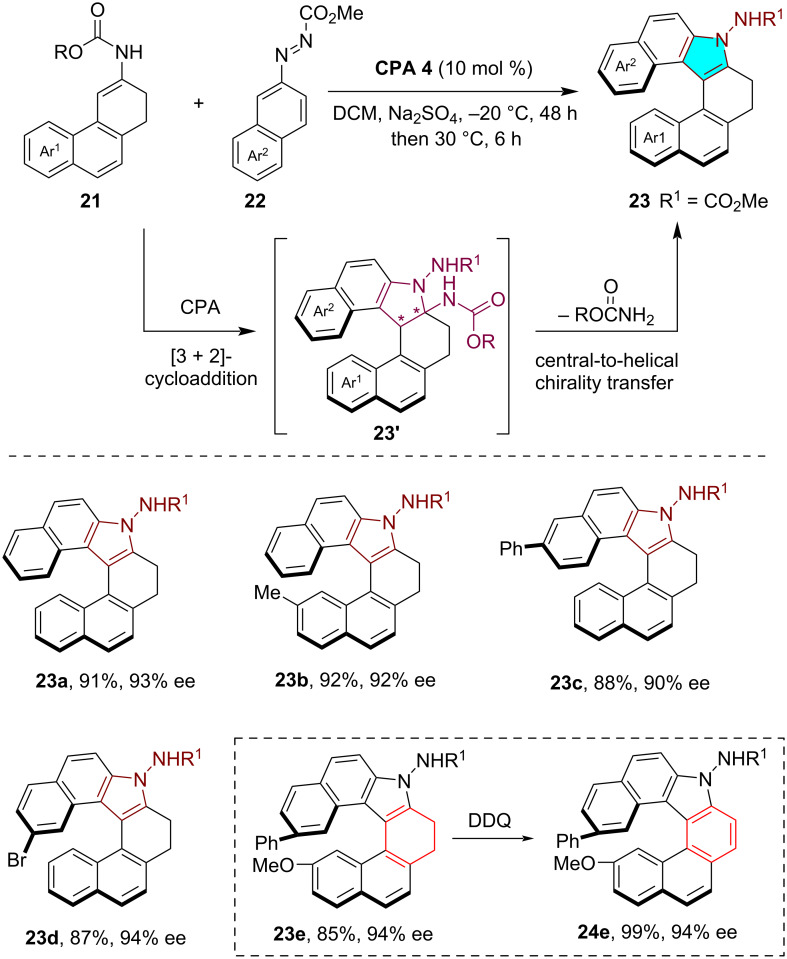
CPA-catalyzed asymmetric synthesis of indolohelicenoids through enantioselective cycloaddition and eliminative aromatization sequence.

In 2024, Zhou, Chen and co-workers disclosed an efficient method for the asymmetric synthesis of indolohelicenoids through a sequential enantioselective annulation, followed by an eliminative aromatization sequence [23]. The CPA-catalyzed asymmetric [3 + 2]-cycloaddition of cycloenecarbamates **21** and carbalkoxy-substituted azonaphthalenes **22** produced the hexacyclic products **23'** with two contiguous stereogenic centers, which then underwent an eliminative aromatization process to yield various indolohelicenoids **23** with excellent enantioselectivity (Scheme 6). The helical chirality of the products **23** was believed to stem from a notably stereospecific central-to-helical chirality conversion process, maintaining high enantioselectivity even when the eliminative aromatization occurred without the CPA catalyst. Notably, indolohelicenoid **23e** could effectively be converted into the fully aromatic indolohelicene **24e** under DDQ-mediated oxidative conditions without compromising the enantiopurity of the compound.

Kinetic resolution stands as one of the most practical and efficient strategies for accessing chiral compounds. Starting from racemic starting materials, this method entails selective conversion of one enantiomer facilitated by a chiral catalyst, yielding enantioenriched products and allowing for the recovery of unreacted substrate with a high level of enantiopurity [24–25]. While CPAs have been extensively utilized in kinetic resolution of centrally chiral [26–28] and axially chiral compounds [29], their application in the kinetic resolution of helically chiral compounds remains largely unexplored.

**Scheme 7:**
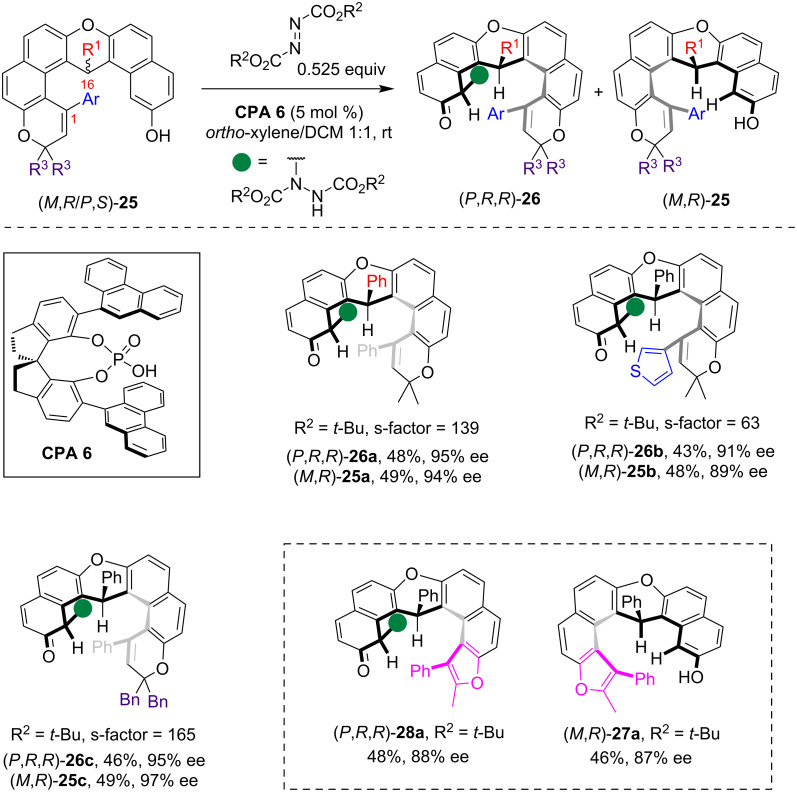
Kinetic resolution of helical polycyclic phenols via CPA-catalyzed enantioselective aminative dearomatization reaction.

In 2024, Liu and co-workers developed an effective method for catalytic kinetic resolution of racemic helical polycyclic phenols through an organocatalyzed enantioselective dearomative amination reaction [30]. The racemic polycyclic phenol derivatives **25**, which exist as single diastereomers featuring both central chirality and helical chirality, were readily prepared through a [3 + 3]-cycloaddition reaction. By employing the CPA-catalyzed asymmetric electrophilic amination reaction with azodicarboxylate on the phenol moiety, efficient kinetic resolution of **25** proceeded to yield both the amination products **26** and the recovered starting material with high enantioselectivity, with an s-factor up to >259 (Scheme 7). Notably, this reaction did not produce the typical arene C–H amination products but instead the dearomative amination products **26**, which is believed to be due to the significant steric hindrance surrounding the amination site that impeded the subsequent aromatization process. Moreover, the terminal ring of the polycyclic phenol substrates was not limited to a pyranoid moiety as helical polycyclic phenols incorporating a furan ring also efficiently yielded both the dearomatized amination product (*P*,*R*,*R*)**-28a** and the recovered enantioenriched phenolic compound (*M*,*R)***-27a** with high enantioselectivity.

**Scheme 8:**
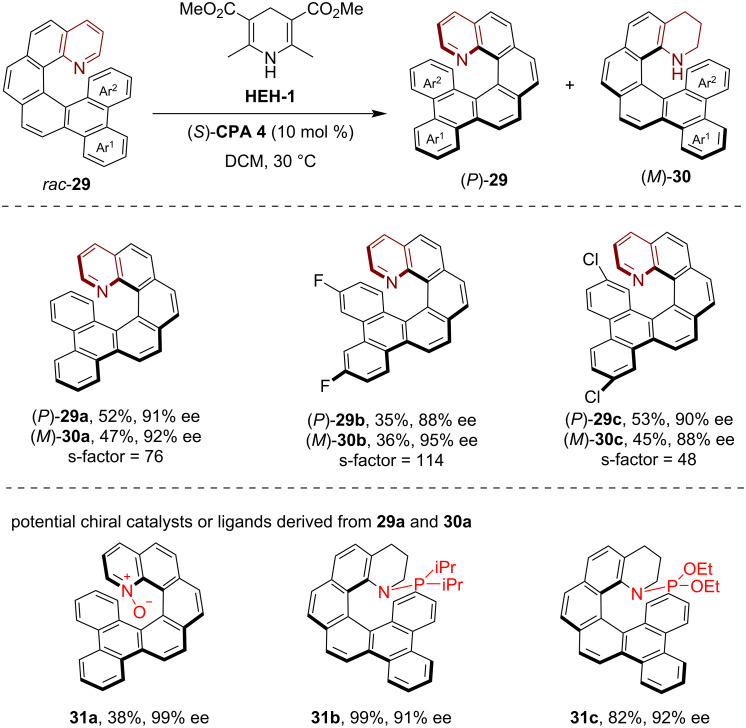
Kinetic resolution of azahelicenes via CPA-catalyzed transfer hydrogenation.

In 2025, Cai, Ji and co-workers reported a practical approach for the kinetic resolution of racemic aza[6]helicenes through CPA-catalyzed asymmetric transfer hydrogenation [31]. Commencing with the readily available racemic pyrido[6]helicene **29**, the CPA-catalyzed asymmetric transfer hydrogenation employing Hantzsch ester **HEH-1** as the reductant afforded both helically chiral tetrahydroquinoline derivatives (*M*)-**30** and the recovered aza[6]helicene starting material (*P*)-**29** with good to high enantioselectivity, achieving an s-factor of up to 121 (Scheme 8). Moreover, by leveraging the synthesized enantioenriched aza[6]helicene **29a** and tetrahydro[6]helicene **30a** as chiral building blocks, a series of helically chiral organocatalysts and ligands could be easily prepared, such as the helically chiral pyridine *N*-oxide **31a** and helically chiral monophosphine ligands **31b**,**c**, whose potential applications in catalytic asymmetric reactions have also been showcased.

### Planar chirality

Planarly chiral cyclophanes, a unique class of macrocyclic compounds featuring planar chirality, can be found in various natural products and are widely utilized in asymmetric catalysis, host–guest chemistry and materials science [32]. These macrocycles typically consist of a substituted aromatic ring and a macrocyclic side chain (*ansa* chain), with the planar chirality arising from the restricted flipping of the substituted aromatic ring caused by steric constraints imposed by the *ansa* chain. Recent advances in asymmetric catalytic synthesis of planarly chiral macrocycles have attracted significant attention, leading to the development of several distinctive strategies, such as (dynamic) kinetic resolution and asymmetric macrocyclizations [33–36].

**Scheme 9:**
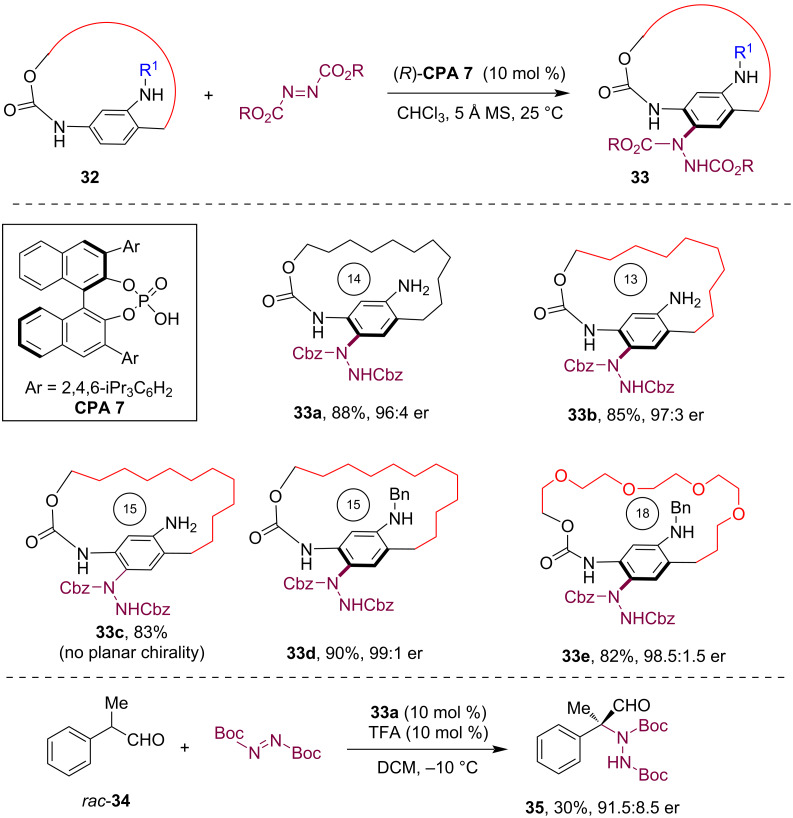
Asymmetric synthesis of planarly chiral macrocycles via CPA-catalyzed electrophilic aromatic amination.

In 2022, our group reported the enantioselective synthesis of planarly chiral macrocycles through a dynamic kinetic resolution approach [15]. Despite bearing an amino group on the phenyl ring, the configuration of the macrocyclic paracyclophane **32** was found to be unstable at room temperature. Consequently, by employing a CPA-catalyzed asymmetric electrophilic amination reaction of the aniline moiety with azodicarboxylates [37–38], the introduction of a bulky hydrazine group restricted the free flipping of the benzene ring, leading to the formation of planar chiral macrocycle **33** with high enantioselectivity (Scheme 9). Substrate scope studies demonstrated the successful construction of planarly chiral macrocycles with 12- to 14-membered *ansa* chains with high enantioselectivity when using NH_2_ as the directing group (see **33a**,**b**). However, extending the *ansa* chain to 15 members led to the loss of planar chirality due to insufficient steric hindrance to restrict the benzene ring flipping (see **33c**). Notably, the use of a bulkier NHBn directing group allowed for the extension of the *ansa* chain to 15–19 members (see **33d**,**e**), while preserving planar chirality and functional group compatibility. Remarkably, the chiral paracyclophane product **33a** could be directly used as a planarly chiral primary amine catalyst in the asymmetric electrophilic amination reaction of aldehyde **34**, which yielded the α-amination product **35** with high enantioselectivity.

**Scheme 10:**
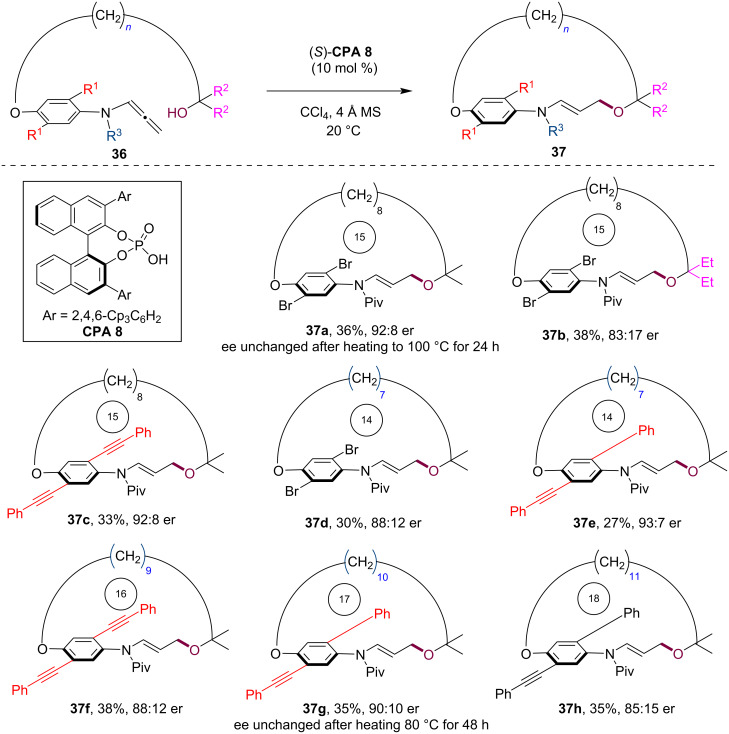
Enantioselective synthesis of planarly chiral macrocycles via CPA-catalyzed macrocyclization.

In 2022, our group disclosed an enantioselective macrocyclization protocol for the asymmetric synthesis of planarly chiral paracyclophanes [39]. Commenced with a macrocyclization precursor **36** featuring both a hydroxy group and an allenamide moiety, the CPA-catalyzed asymmetric intramolecular addition led to the successful construction of planarly chiral macrocycles **37** (Scheme 10). This method demonstrated broad substrate compatibility, accommodating sterically demanding dibromo and various dialkynyl substitutions on the phenyl ring. A series of planarly chiral macrocycles with *ansa* chains ranging from 15 to 18 members was synthesized with good to high enantioselectivity, albeit with moderate yield. Significantly, thermal stability studies demonstrated high configurational stability of these planarly chiral macrocycles, a critical feature that enhances their potential for future utility.

**Scheme 11:**
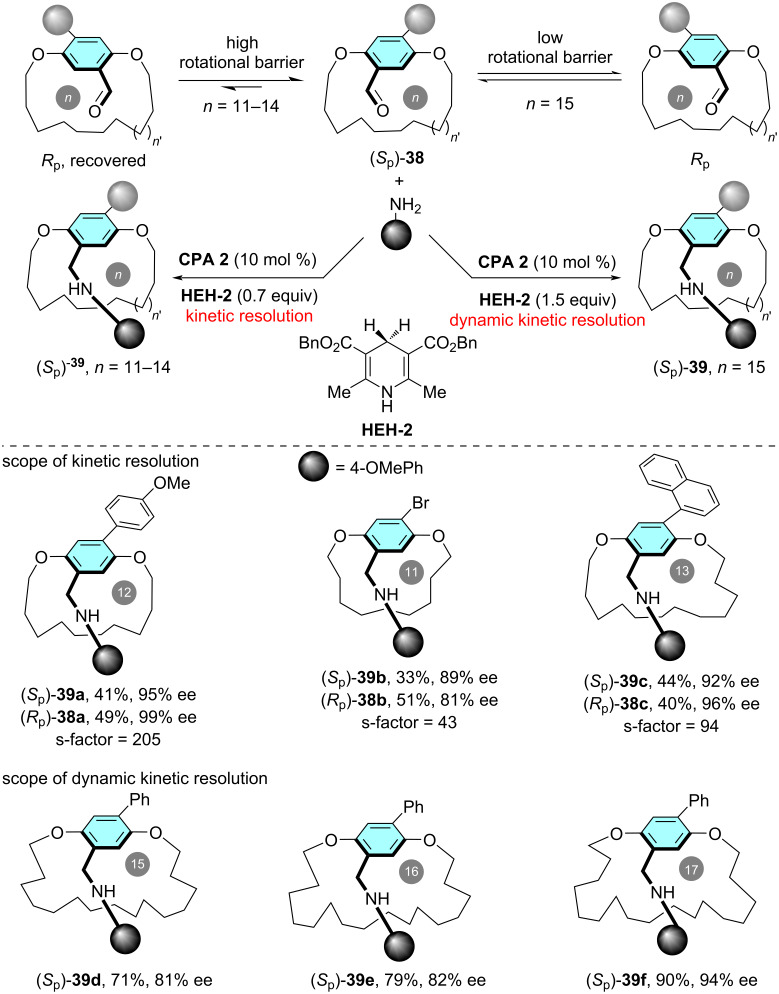
(Dynamic) kinetic resolution of planarly chiral paracyclophanes via CPA-catalyzed asymmetric reductive amination.

In 2023, Zhao and co-worker reported the asymmetric synthesis of planarly chiral paracyclophanes through either catalytic kinetic resolution or dynamic kinetic resolution [40]. The authors designed and synthesized a series of benzaldehyde-containing macrocyclic cyclophanes **38**. Therein, they achieved the construction of planar chirality through CPA-catalyzed asymmetric reductive amination with arylamines using Hantzsch ester **HEH-2** as the hydrogen transfer reagent (Scheme 11). Notably, when starting from macrocyclic substrates featuring relatively shorter *ansa* chains (11–14 members, see **38a–c**), highly efficient kinetic resolution was achieved, resulting in both recovered (*R*_p_)-**38** and reductive amination products (*S*_p_)-**39** with high enantioselectivity. Conversely, employing macrocyclic paracyclophane with longer *ansa* chains (≥15 members) enabled efficient dynamic kinetic resolution due to the instable planar chirality of the substrates, which produced the planarly chiral macrocycles with high yield and enantioselectivity (up to 98% yield and 99% ee).

**Scheme 12:**
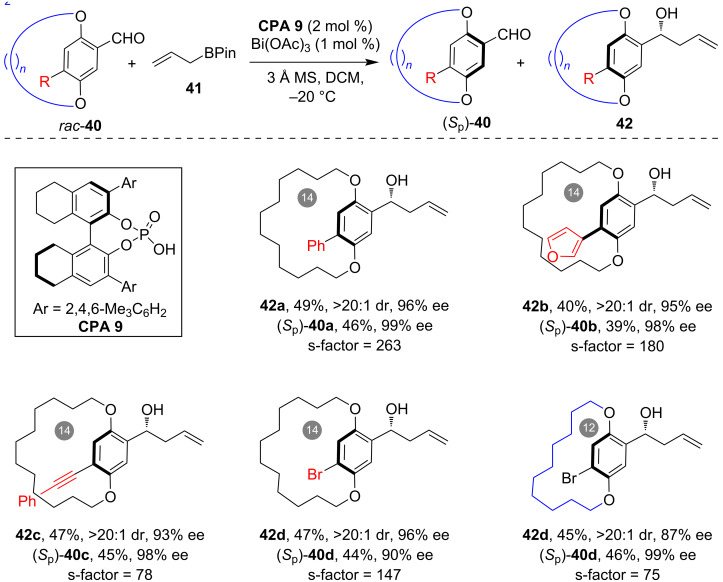
Kinetic resolution of macrocyclic paracyclophanes through CPA/Bi-catalyzed asymmetric allylation.

In 2025, Li and co-workers utilized analogous racemic benzaldehyde-containing paracyclophanes as substrates and accomplished their efficient kinetic resolution through catalytic asymmetric allylation [41]. Employing CPA/Bi(OAc)_3_ as a combined catalyst, the asymmetric allylation of racemic **40** with allylboronic acid pinacol ester (**41**) led to efficient kinetic resolution, yielding the recovery of (*S*_p_)-**40** with high enantiopurity (Scheme 12). Notably, the allylation products **42**, possessing both planar chirality and central chirality, were produced with high enantioselectivity and diastereoselectivity. Previously, they have been challenging to access in an asymmetric one-step reaction. A range of paracyclophanes with diverse substitutions, including aryl, heteroaryl, alkynyl and bromo substitutions, along with a varying length of the *ansa* chain, were found to be amenable to this method, resulting in kinetic resolution with an exceptional performance.

**Scheme 13:**
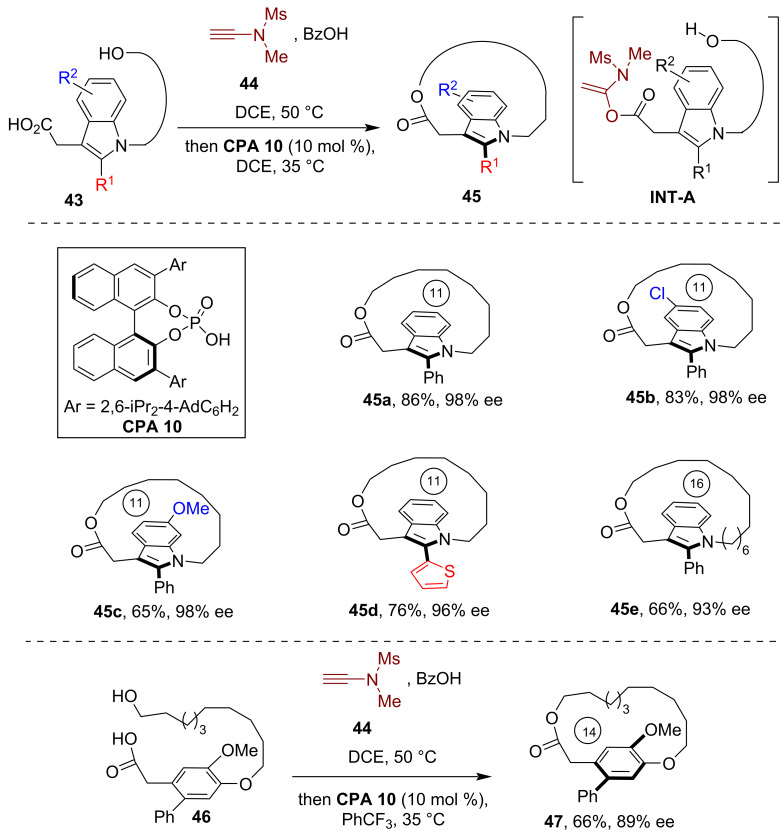
Enantioselective synthesis of planarly chiral macrocycles via CPA-catalyzed coupling of carboxylic acids with alcohols via ynamide mediation.

In 2025, Zhou and co-workers disclosed the asymmetric synthesis of planarly chiral macrocycles via CPA-catalyzed atroposelective macrocyclization [42]. The authors devised and prepared a series of indole-based hydroxy-substituted carboxylic acid substrates **43** which, upon treatment with ynamide **44**, yielded the vinyl acetate intermediate **INT-A** (Scheme 13). Subsequently, the one-pot CPA-catalyzed intramolecular esterification of this intermediate afforded the planarly chiral macrocycles **45** with good yield and high enantioselectivity. Investigations of the substrate scope revealed the compatibility of the method with various substitutions on the indole moiety and modifications to the length of the *ansa* chain, which produced planarly chiral macrocycles with up to 99% ee. In addition, this method was successfully employed for the catalytic asymmetric synthesis of planarly chiral macrocyclic paracyclophane **47** from the corresponding hydroxy-substituted carboxylic acid substrate **46**. Notably, the authors also demonstrated the application of this method in the enantioselective synthesis of axially chiral C–N and N–N atropisomers, highlighting the versatility of this method in the asymmetric synthesis of diverse chiral molecular structures.

**Scheme 14:**
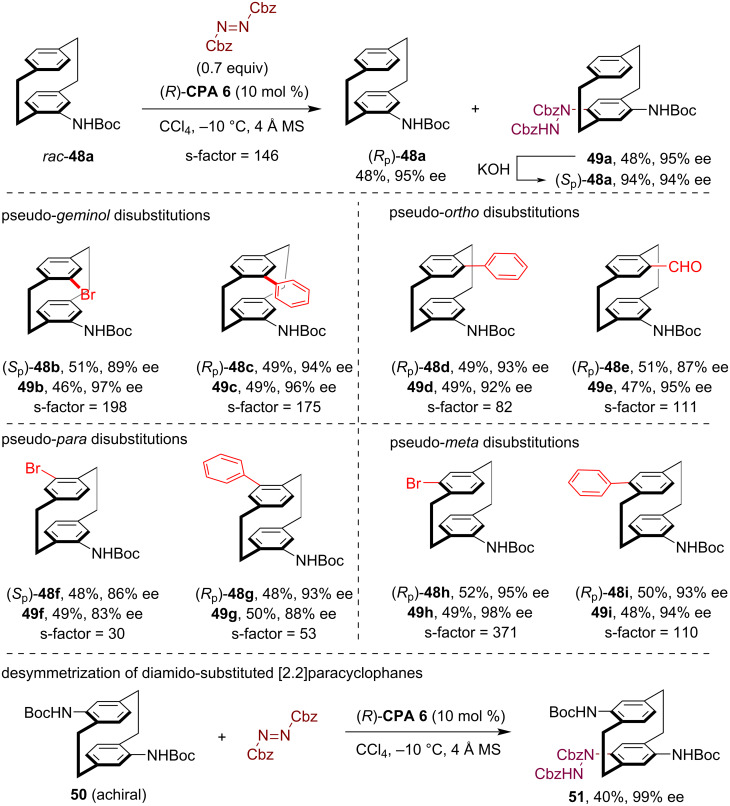
Kinetic resolution of substituted amido[2.2]paracyclophanes via CPA-catalyzed asymmetric electrophilic amination.

Substituted [2.2]paracyclophanes represent another class of conformationally rigid, planarly chiral molecules, which have emerged as versatile scaffolds for developing chiral catalysts, ligands and functional materials. In 2023, our group reported the first catalytic kinetic resolution of racemic amido[2.2]paracyclophanes through a CPA-catalyzed asymmetric aromatic amination reaction [43]. Treating the racemic *N-*Boc-substituted [2.2]paracyclophane **48a** with dibenzyl azodicarboxylate (0.7 equiv) in the presence of **CPA 6** (10 mol %) led to efficient kinetic resolution, yielding both the *para*-C–H amination product **49a** and the recovered starting material (*R*_p_)-**48a** with high enantioselectivity (Scheme 14). Notably, subjecting **49a** to strongly basic conditions resulted in dehydrazidation to give (*S*_p_)-**48a**, and thus enabling facile access to both amido[2.2]paracyclophane enantiomers. Moreover, this method demonstrated broad substrate generality, which enabled the efficient kinetic resolution of various disubstituted amido[2.2]paracyclophanes, including the pseudo*-geminal*- (see **48b**,**c**), pseudo-*ortho*- (see **48d**,**e**), pseudo-*meta*- (**48f**,**g**) and pseudo-*para*-substituted ones (see **48h**,**i**). Furthermore, this method could also be utilized for the enantioselective desymmetrization of achiral diamido-substituted [2.2]paracyclophane substrate **50**, delivering the C–H amination product **51** with excellent enantioselectivity (99% ee).

### Inherent chirality

The concept of inherent chirality was first coined by Böhmer and co-workers in 1994 to describe the chirality originating from the asymmetric arrangement of achiral substituents within calixarene frameworks [44]. This term was later extended to encompass other conformationally rigid chiral molecules that do not fit into conventional categories of central, axial, planar or helical chirality, such as saddle-shaped, medium-sized cyclic compounds [45] and others [46]. These structurally distinct chiral molecules have received considerable research attention due to their broad range of potential applications in chiral recognition, sensing and asymmetric catalysis. However, achieving the catalytic asymmetric synthesis of these inherently chiral molecules remains highly challenging owing to their unique three-dimensional structures and relatively large size [47–48].

**Scheme 15:**
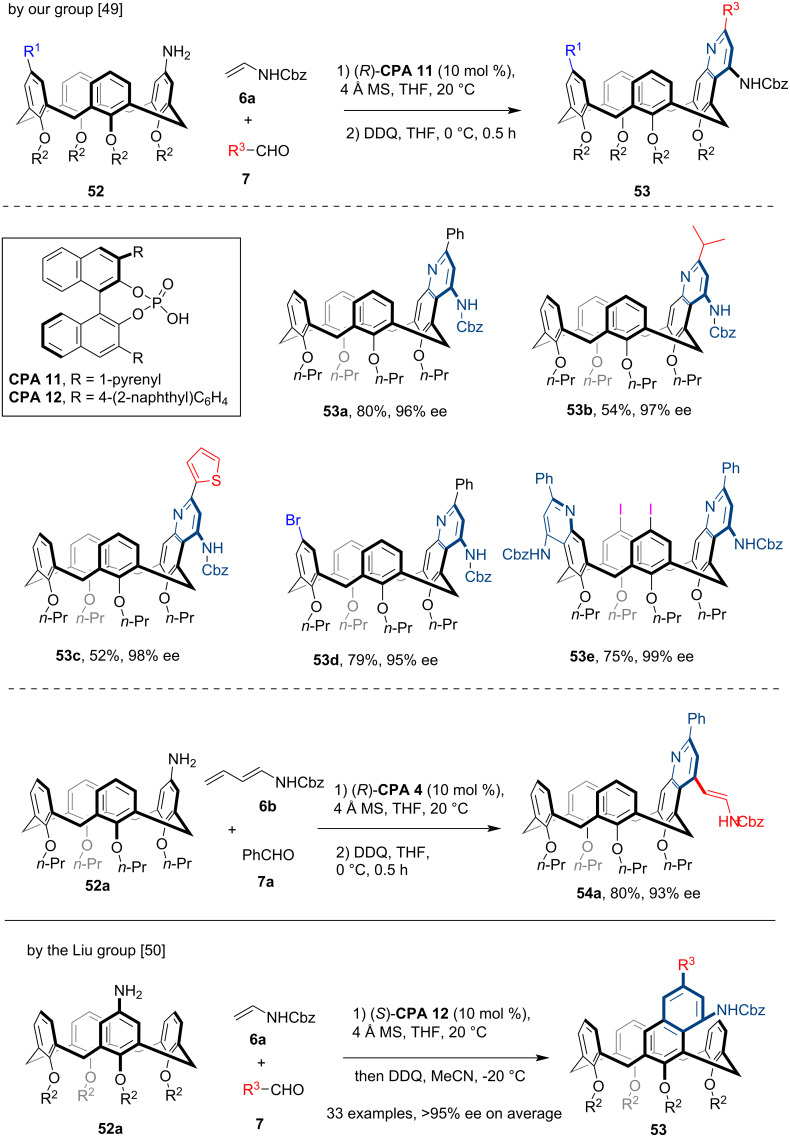
Enantioselective synthesis of inherently chiral calix[4]arenes via sequential CPA-catalyzed Povarov reaction and aromatization.

In 2024, both our group [49] and the Liu group [50] independently reported the asymmetric synthesis of inherently chiral calix[4]arenes through an enantioselective desymmetrization strategy. Starting from the achiral aniline-containing calix[4]arenes **52**, we employed the **CPA 11**-catalyzed asymmetric Povarov reaction [51] with enamide **6a** and various aldehydes **7** to break the symmetry of substrates **52**, which was followed by a one-pot oxidative aromatization mediated by DDQ to yield the quinoline-containing inherently chiral calix[4]arenes **53** (Scheme 15). Notably, the prochiral calix[4]arenes bearing a disubstitution pattern on the 1,3-phenyl rings (see **53d**) or 1,3-diamino substitution (see **53e**) on the calix[4]arene scaffold were also amenable to this method, which yielded a series of structurally diverse novel quinoline-containing inherently chiral calix[4]arenes. Moreover, by using **CPA 4** as the optimal catalyst, the sequential asymmetric Povarov reaction of **52a** with dienamide **6b** and benzaldehyde **7a**, followed by oxidative aromatization, led to the formation of enamide-substituted, quinoline-containing inherently chiral calix[4]arene **54a**, whose enamide moiety could undergo diverse derivatizations. Analogously, the Liu group achieved the asymmetric synthesis of inherently chiral quinoline-containing calix[4]arenes **53** through the same approach, using (*S*)-**CPA 12** as the optimal catalyst.

**Scheme 16:**
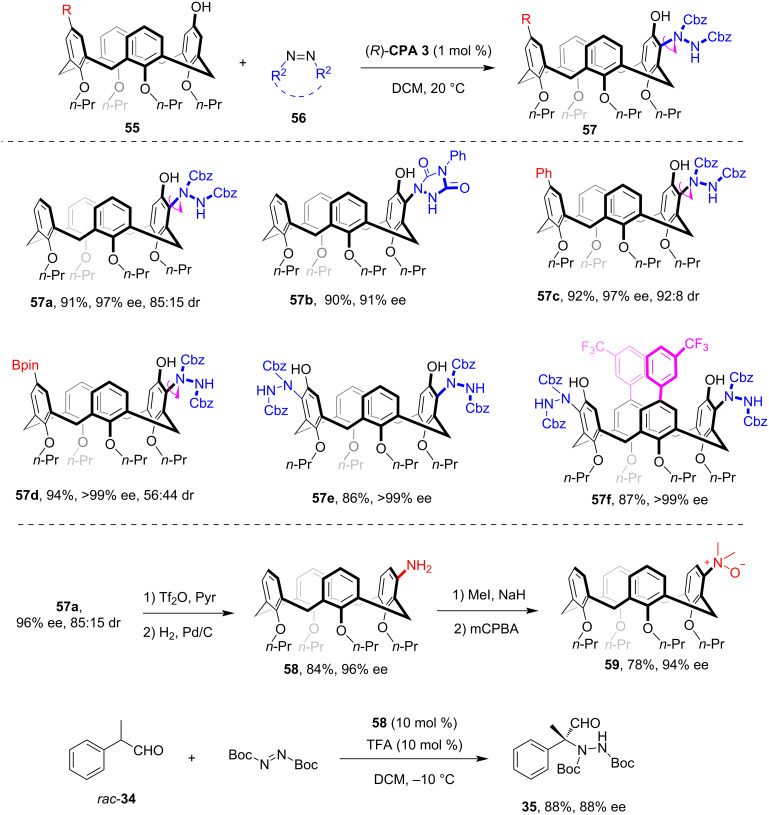
Asymmetric synthesis of inherently chiral calix[4]arenes via CPA-catalyzed aminative desymmetrization.

In 2025, our group presented another example of an asymmetric synthesis of inherently chiral calix[4]arenes using a CPA-catalyzed enantioselective desymmetrization strategy [52]. Commencing with phenol-containing prochiral calix[4]arenes **55**, the **CPA 3**-catalyzed asymmetric *ortho*-C–H amination with electrophilic azo reagents **56** effectively broke the symmetry of the substrate, leading to the formation of inherently chiral calix[4]arenes **57** with high enantioselectivity (Scheme 16). Notably, with the use of acyclic azodicarboxylate as amination reagent, the products exhibited both inherent chirality and intriguing C–N axial chirality (see **57a**). This method demonstrates excellent substrate compatibility, accommodating various calix[4]arenes with 1,3-phenyl ring disubstitution patterns (see **57c**,**d**) and diphenol-containing calix[4]arenes (see **57e**,**f**). The aminated chiral calix[4]arene products underwent diverse derivatizations due to the abundance of functional groups present. Moreover, the potential applications of these unique inherently chiral calix[4]arenes have also been showcased. For instance, facile derivatizations of **57a** afforded the inherently chiral *meta*-amino-substituted calix[4]arene **58** and the corresponding aniline *N-*oxide **59**. Our study suggested that inherently chiral calix[4]arene **58** could successfully be used as a chiral organocatalyst in the asymmetric amination of aldehyde **34**, whereas inherently chiral aniline *N-*oxide **59** showed promise in the chiral recognition of mandelic acid.

**Scheme 17:**
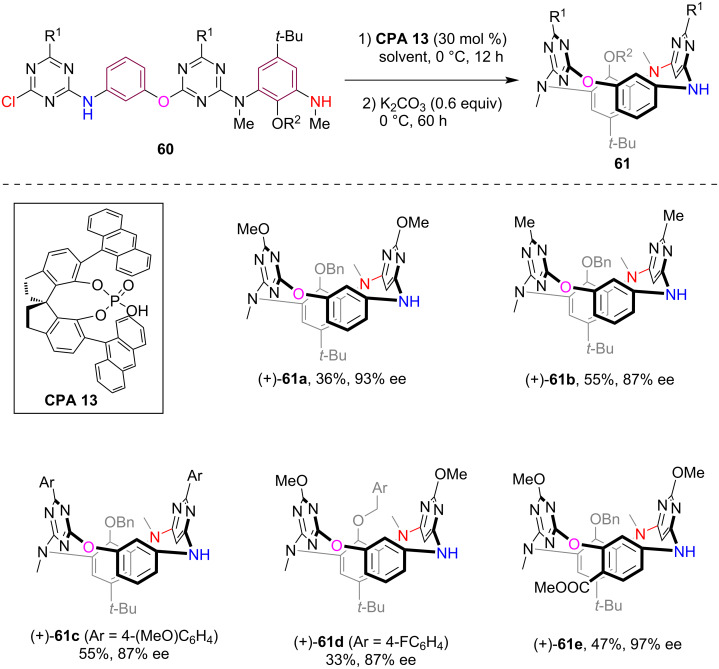
Asymmetric synthesis of chiral heterocalix[4]arenes via CPA-catalyzed intramolecular S_N_Ar reaction.

In 2024, Tong, Wang and co-workers disclosed an efficient method for synthesizing inherently chiral heterocalix[4]arenes through an asymmetric macrocyclization strategy [53]. Starting from the linear precursor **60** bearing two triazine moieties, the intramolecular S_N_Ar reaction catalyzed by **CPA 13** (30 mol %) led to macrocyclization, which produced the inherently chiral N_3_,O*-*calix[2]arene[2]triazines **61** with high enantioselectivity, albeit in moderate yield (Scheme 17). The addition of K_2_CO_3_ after 12 hours improved the enantioselectivity of this reaction by scavenging the HCl produced during the S_N_Ar reaction, which was believed to potentially promote the nonenantioselective background macrocyclization reaction. Notably, these inherently chiral heterocalix[4]arenes displayed a distinctive 1,3-alternate conformation, notably differing from the typical cone conformation of the conventional calix[4]arenes. Moreover, unlike previously documented examples, the inherent chirality of these products arises from the difference of just one heteroatom (O and NH) in the linking positions of the heterocalix[4]arenes, which may pave new avenues for designing and synthesizing inherently chiral macrocycles.

**Scheme 18:**
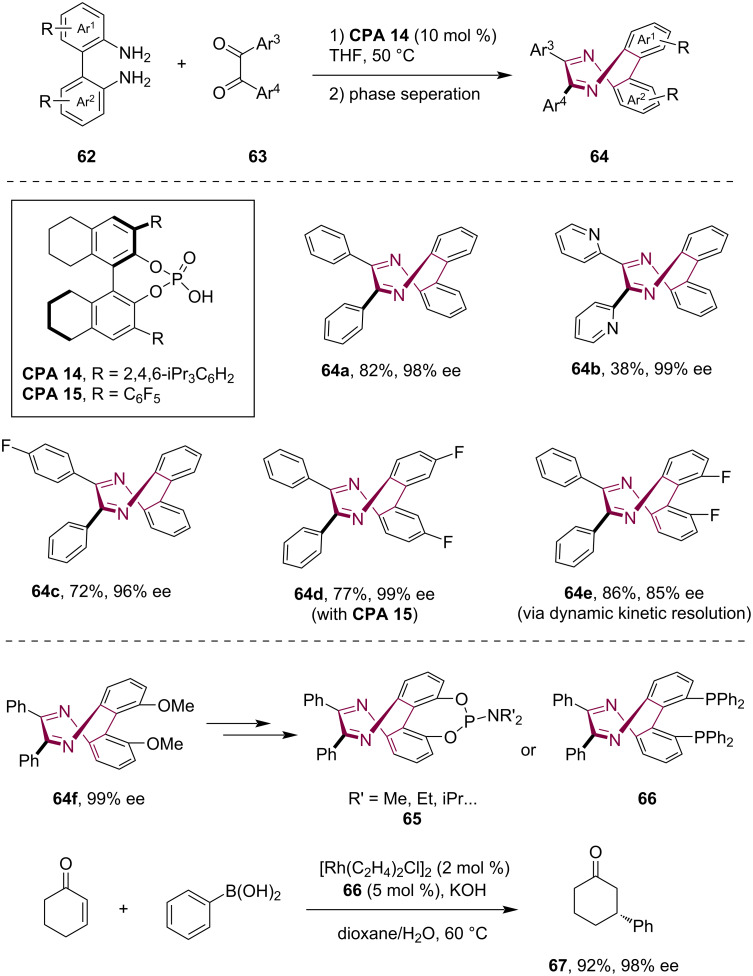
Enantioselective synthesis of inherently chiral DDDs via CPA-catalyzed cyclocondensation.

Cyclic molecules smaller than calix[4]arenes that possess a rigid nonplanar conformation can also exhibit inherent chirality. In 2023, Luo, Zhu and co-workers reported the efficient asymmetric synthesis of inherently chiral eight-membered N*-*heterocycle 6,7-diphenyldibenzo[*e*,*g*][1,4]diazocines (DDDs), which displayed a rigid saddle-shaped configuration [16]. Starting from readily available [1,1'-biphenyl]-2,2'-diamines **62** and benzyl compounds **63**, the asymmetric cyclocondensation between these two components enabled by CPA catalysts yielded the inherently chiral DDDs **64** with good to high enantioselectivity (Scheme 18). While a number of reactions did not initially yield satisfactory enantioselectivity, facile phase separation during the workup process removed the less soluble racemic products, which resulted in the isolation of chiral products with exceptional enantiopurity. Moreover, this method accommodated [1,1'-biphenyl]-2,2'-diamines **62** with *ortho*-substitutions, which underwent either dynamic kinetic resolution or kinetic resolution to produce chiral substituted DDD products **64e**. Moreover, the authors showcased the facile derivatization of dimethoxy-substituted chiral DDD **64f** into various DDD-based chiral ligands, such as the phosphoramidites **65**, phosphoric acid as well as monophosphine ligands and diphosphine ligands **66**. Notably, the applications of these novel inherently chiral ligands have been explored. For example, they demonstrated excellent enantioselectivity control in some asymmetric reactions, such as the Rh/diphosphine ligand **66**-catalyzed asymmetric addition reaction between cyclic enone and arylboronic acid.

**Scheme 19:**
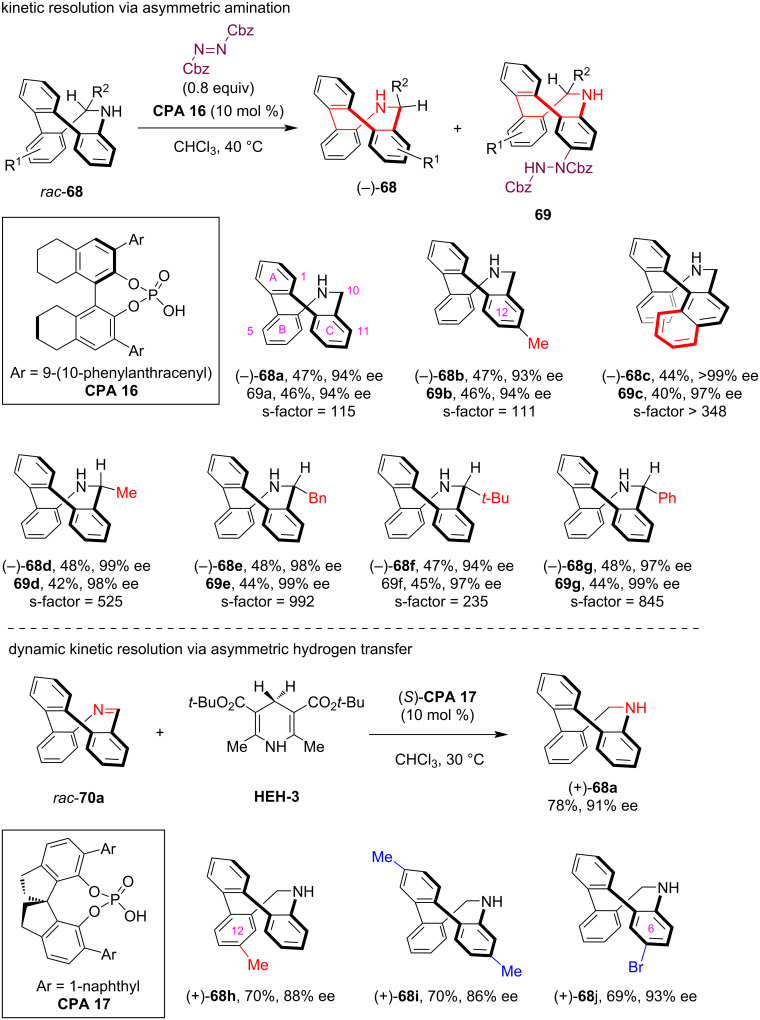
Asymmetric synthesis of saddle-shaped inherently chiral 9,10-dihydrotribenzoazocines via CPA-catalyzed (dynamic) kinetic resolution.

In 2024, our group reported the catalytic asymmetric synthesis of saddle-shaped inherently chiral 9,10-dihydrotribenzoazocines via CPA-catalyzed kinetic resolution and dynamic kinetic resolution strategies [54]. By leveraging the reactivity of the aniline moiety in 9,10-dihydrotribenzoazocines **68**, the **CPA 16**-catalyzed enantioselective *para-*selective C–H amination reaction with dibenzyl azodicarboxylate (0.8 equiv) resulted in efficient kinetic resolution, which yielded both the C–H amination product **69** and recovered (+)-**68** with high enantioselectivity (see **68a**–**c**, Scheme 19). Moreover, this method was also applicable to the kinetic resolution of racemic 10-substituted 9,10-dihydrotribenzoazocines featuring both inherent and central chirality, delivering excellent kinetic resolution performance (see **68d**–**g**). During our studies, we serendipitously found that the imine-containing eight-membered azaheterocycles **70**, derived from the oxidative dehydrogenation of **68**, displayed unexpectedly low configurational stability. Consequently, we developed a more efficient dynamic kinetic resolution protocol for the asymmetric synthesis of inherently chiral **68**. This method involved the **CPA 17**-catalyzed asymmetric hydrogen transfer reaction of racemic **70** using Hantzsch ester **HEH-3** as the reductant, which enabled the asymmetric synthesis of some inherently chiral substituted 9,10-dihydrotribenzoazocines that had been challenging to access through the aminative dearomatization method (see **68h**,**i**).

**Scheme 20:**
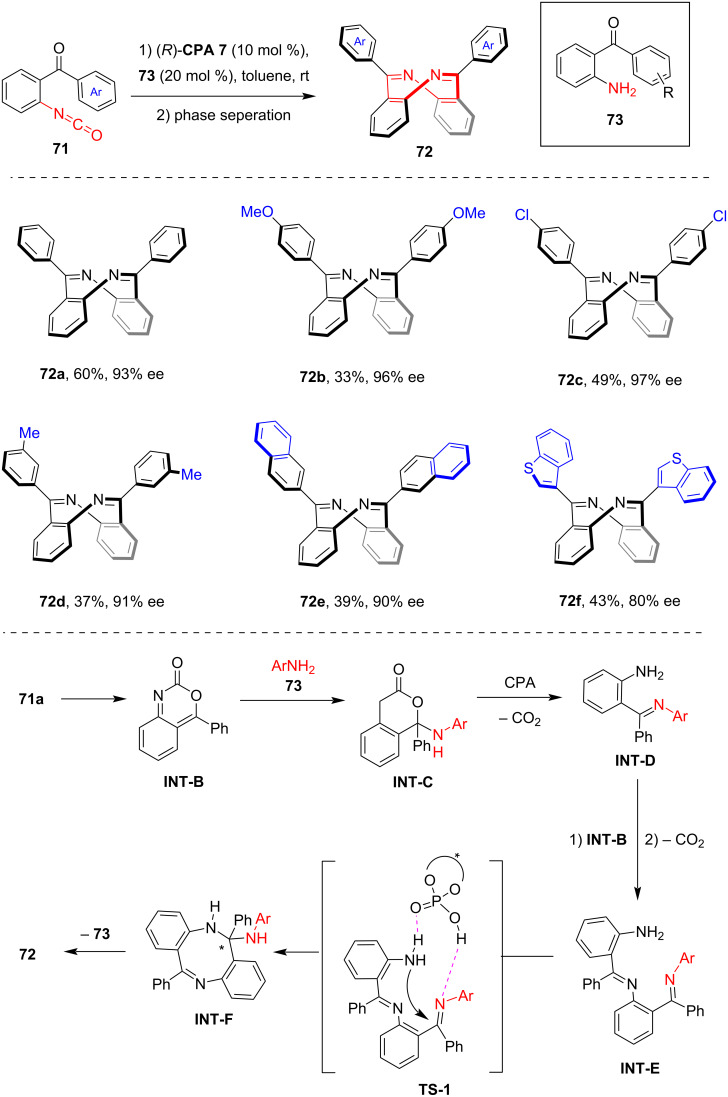
Enantioselective synthesis of inherently chiral saddle-shaped dibenzo[*b*,*f*][1,5]diazocines via CPA-catalyzed dimerization of 2-acylbenzo isocyanates.

In 2024, our group reported a convenient method for the asymmetric synthesis of saddle-shaped inherently chiral dibenzo[*b*,*f*][1,5]diazocines **72** via CPA catalysis [55]. In the presence of **CPA 7** (10 mol %) and the corresponding 2-acylaniline **73** (20 mol %) as co-catalysts, the asymmetric dimerization of 2-acylbenzo isocyanates **71** allowed access to inherently chiral eight-membered azaheterocycles **72** with moderate to good enantioselectivity, along with the release of CO_2_ (Scheme 20). While the enantioselectivity using certain substrates was initially unsatisfactory, simple phase separation significantly enhanced the enantiopurity of the products by removing the less soluble racemic products. Detailed studies were conducted to explore the reaction mechanism, focusing specifically on the role of the 2-acylanilines **73** as co-catalysts. Based on the experimental results and previous research, a plausible mechanism was proposed. Isomerization of substrates **71** yielded the cyclic intermediate **INT-B**, which then underwent addition with aniline co-catalyst **73** to form **INT-C**. The CPA-enabled release of CO_2_ from **INT-C** yielded the imine-containing intermediate **INT-D**, which underwent iterative addition with **INT-B**, followed by release of CO_2_ to afford **INT-E**. The CPA-catalyzed cyclization of **INT-E** through the dual hydrogen bonding activation transition state **TS-1** afforded the eight-membered heterocycle **INT-F** with a stereogenic center. Through the elimination of aniline **73**, the saddle-shaped dibenzo[1,5]diazocine **72** was produced via a central-to-inherent chirality transfer process. Notably, while only the amino group of the co-catalysts was shown to engage in the catalytic cycle, the 2-acyl group of **73** was believed to participate in hydrogen bonding interactions with the substrate and the CPA catalyst, playing additional crucial roles.

**Scheme 21:**
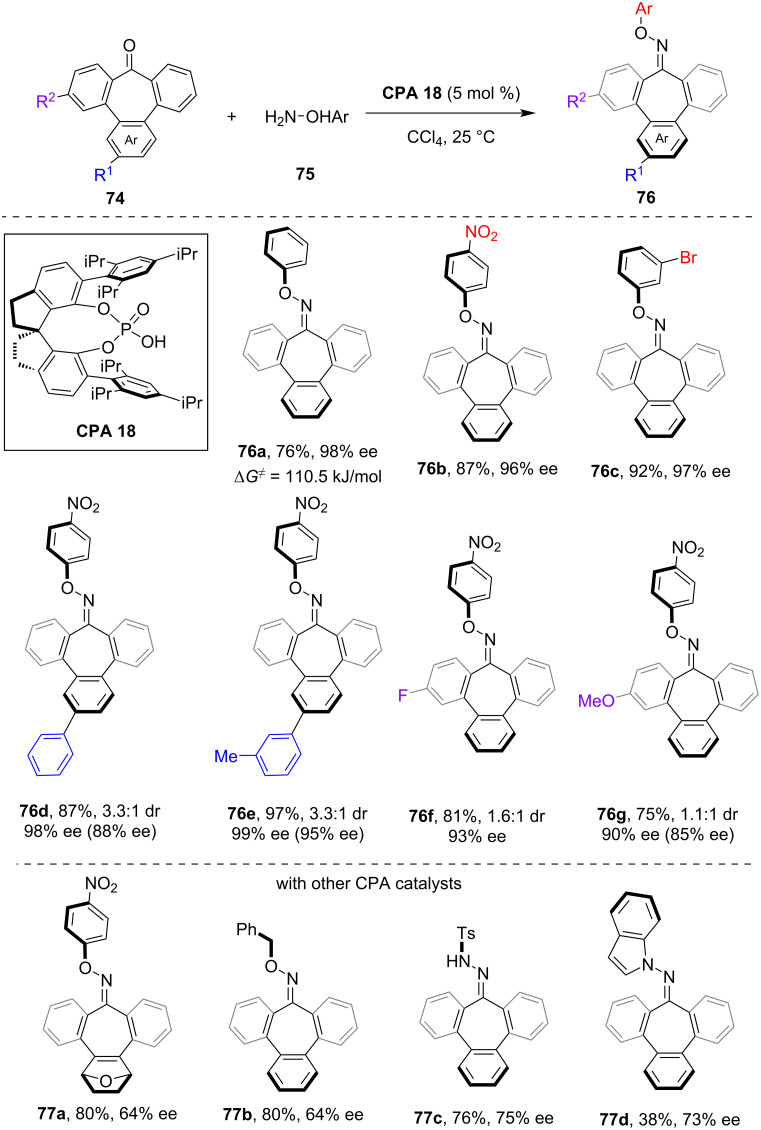
Enantioselective synthesis of inherent chiral 7-membered tribenzocycloheptene oximes via CPA-catalyzed condensation.

In addition to various saddle-shaped eight-membered azaheterocycles, conformationally rigid seven-membered cyclic compounds can also exhibit inherent chirality. In 2017, Antilla et al. developed the CPA-catalyzed asymmetric condensation of 4-substituted cyclohexanones with *O*-arylhydroxylamines, which yielded axially chiral cyclohexylidene oxime ethers with high enantioselectivity [56]. In 2024, through the utilization of this method, Liu and co-workers disclosed the enantioselective synthesis of inherently chiral 7-membered tribenzocycloheptene oximes **76** through **CPA 18**-catalyzed asymmetric condensation between 7-membered cyclic ketones **74** and hydroxylamines **75** (Scheme 21) [57]. High to excellent yield and enantioselectivity were achieved for the inherently chiral products when using a range of substituted arylhydroxylamines (see **76a**–**c**). The racemization barrier of the product **76a** was determined to be 110.5 kJ/mol, which suggested the relative instability of the configuration of these structurally unique products compared to the eight-membered inherently chiral compounds. Moreover, unsymmetrical substituted cyclic ketones **74** were investigated under the standard conditions, which produced a pair of diastereomers with poor diastereoselectivity while maintaining high enantioselectivity for both diastereomers (see **76d**–**g**). Furthermore, the authors have investigated the asymmetric condensation using other seven-membered cyclic ketones (see **77a**) as well as the coupling with alkylhydroxylamine (see **77b**), tosylhydrazide (see **77c**) and *N-*aminoindole (see **77d**), which all produced the inherently chiral products with moderate to good enantioselectivity, albeit requiring the use of different CPA catalysts.

## Conclusion

The increasing number of applications of non-centrally-chiral molecules, including helically chiral, planarly chiral and inherently chiral molecules across diverse research fields, has spurred considerable research focus toward the catalytic asymmetric synthesis of these unique chiral molecules. While methods for the asymmetric synthesis of these chiral molecules remain relatively underexplored compared to the enantioselective synthesis of centrally and axially chiral compounds, significant progress has been made in these fields in recent years. Among numerous chiral catalysts, CPAs have emerged as key players in the asymmetric synthesis of these structurally unique chiral molecules, owing to their diverse catalytic abilities, precise stereoselectivity control and mild reaction conditions. In this Review, we systematically summarized the advancements in the CPA-catalyzed asymmetric synthesis of helically chiral, planarly chiral and inherently chiral molecules. Various CPA-catalyzed reactions, such as cyclizations, aromatic substitutions and condensations, along with asymmetric synthesis strategies, such as enantioselective desymmetrization and (dynamic) kinetic resolution, have been employed for the asymmetric construction of these chiral elements.

Despite remarkable progress and significant potential in the CPA-catalyzed asymmetric synthesis of these unique chiral molecules, some current limitations and challenges still need to be addressed, particularly enhancing the efficiency of the methods and expanding the structural diversity of the products. Firstly, the chiral products generated through CPA-catalyzed methods are still relatively simple. For instance, in terms of helically chiral helicenes, typically, only the relatively shorter [5]helicenes have been produced, while the more complex, longer helicenes and multihelicenes have not yet been successfully synthesized through CPA-catalyzed asymmetric methods. Secondly, asymmetric synthetic strategies based on presynthesized three-dimensional molecular structures are commonly employed, such as enantioselective desymmetrization and (dynamic) kinetic resolution. While these strategies have proven effective, the efficiency of these methods may not be considered highly satisfactory due to the requirement to prepare relatively complex substrates. Therefore, there is a high demand for the development of more efficient asymmetric methods through which molecular structures can be directly constructed while achieving high enantioselectivity. Overall, with the recent rapid advancements of CPA catalysis, along with the utilization of CPA catalysts in asymmetric radical chemistry, transition metal-catalyzed reactions and photoredox chemistry, we envision that CPA catalysts will continue to play a central role in the future asymmetric synthesis of helically chiral, planarly chiral and inherently chiral molecules.

## Data Availability

Data sharing is not applicable as no new data was generated or analyzed in this study.
